# MicroRNA‐205‐5p: A potential therapeutic target for influenza A

**DOI:** 10.1111/jcmm.17615

**Published:** 2022-11-20

**Authors:** Yanyan Bao, Yujing Shi, Lirun Zhou, Shuangrong Gao, Rongmei Yao, Shanshan Guo, Zihan Geng, Lei Bao, Ronghua Zhao, Xiaolan Cui

**Affiliations:** ^1^ Institute of Chinese Materia Medica China Academy of Chinese Medical Sciences Beijing China; ^2^ Institute of Traditional Chinese Medicine Tianjin University of Traditional Chinese Medicine Tianjin China

**Keywords:** influenza a virus, MicroRNA‐205‐5p, nucleoprotein, therapeutic target

## Abstract

We are committed to finding host targets for influenza A therapeutics. The nucleoprotein (NP) plays an important role in influenza A virus replication and is an indispensable part of viral transcription and replication. Exploring endogenous substances that can modulate NP is critical for finding host targets. MicroRNAs (miRNAs, miR) are a novel class of powerful, endogenous gene expression regulators. Herein, we used miRanda to analyse the base complementarity between the *NP* gene and the 14 host miRNAs reported previously by us. MiRanda predicted that miR‐431‐5p, miR‐744‐3p and miR‐205‐5p could complement the *NP* gene. To understand the effect of these miRNAs on NP expression, we co‐transfected 293 T cells with *NP* gene sequence containing above miRNAs binding site or full sequence of *NP* gene (transfected into pmirGlo or pcDNA3.1 vectors, respectively), and mimics of miR‐205‐5p, miR‐431‐5p and miR‐744‐3p. Dual luciferase reporter gene or Western blotting assays confirmed that miR‐205‐5p and miR‐431‐5p inhibit NP expression by binding with the miRNA binding site of *NP* gene. Further, we infected Mouse Lung Epithelial (MLE‐12) cells overexpressing miR‐205‐5p and miR‐431‐5p with influenza A virus and performed Western blotting to examine NP expression. We found that NP expression was significantly reduced in MLE‐12 cells overexpressing miR‐205‐5p during influenza A infection. The miR‐205‐5p overexpression‐induced inhibition of influenza A replication could be attributed to the inhibition of NP expression. Further, we administered oseltamivir and Jinchai Antiviral Capsules (JC, an anti‐influenza Chinese medicine) to influenza A virus‐infected MLE‐12 cells and mice. We found that miR‐205‐5p was significantly decreased increased in infected cells and lung tissues, and oseltamivir and JC could up‐regulate miR‐205‐5p. In conclusion, we provide new evidence that miR‐205‐5p plays a role in regulating viral NP protein expression in combating influenza A and may be a potential target for influenza A therapy.

## 1 INTRODUCTION

The influenza A virus has a wide host range, strong infectivity, rapid spread, and is prone to mutation.^1^ Due to the lack of completely effective prevention and treatment methods, Type A influenza is still a global health threat.^2^ Influenza A virus is a major cause of morbidity and a long‐standing lurking virulent strain that can cause local outbreaks or global epidemics in a short time.^3^ Therefore, it is extensively researched by virologists. Due to the wide application of anti‐influenza A virus drugs, the drug resistance of influenza A virus is increasing day by day.^4^, ^5^ Therefore, it is urgent to develop new antiviral drugs and find new potential targets. The host target is not easy to mutate and is more conducive to the durability of the drug, which has become a research hotspot of researchers in recent years.^6^, ^7^, ^8^

The nucleoprotein (NP) plays an important role in influenza A virus replication; it is an indispensable part of viral transcription and replication.^9^, ^10^, ^11^ NP expression inhibition can block virus replication. Therefore, it is very important to explore the endogenous substances that can inhibit the NP for finding host targets.

MicroRNAs (miRNAs, miR) are a class of naturally occurring small and non‐coding RNA molecules. They bind to the 3′ untranslated regions (3′‐UTR) of target mRNAs to either block the translation or initiate the transcript degradation.^12^ In 2005, scientists discovered for the first time that host miRNA exhibits antiviral function.^13^ Since then, host miRNA and viral gene interactions have become a new research hotspot in the field of miRNA and host antiviral defence. miR‐323, miR‐491 and miR‐654 target influenza A virus base polymerase 1 gene (*PB1*) and inhibit the in vitro replication of H1N1 influenza virus in Madin‐Darby Canine Kidney (MDCK) cells.^14^ Let‐7c aberrantly expresses in influenza A virus infected human lung epithelial cells (A549) and targets the matrix protein 1 gene (*M1*) in influenza A virus and inhibits influenza A virus replication in A549 cells, plays a role in protecting host cells from the virus.^15^ However, thus far, miRNAs have not been systematically targeted for anti‐influenza A viral therapy.

Based on the above reasons, host miRNAs are very likely to become novel drug therapeutic targets for influenza A. In this study, we aimed to mine host miRNAs that could not only inhibit NP expression in host cells but also be regulated by anti‐influenza A drugs. As a result, miR‐205‐5p stood out from the 14 candidate miRNAs and became a powerful endogenous candidate for influenza A therapeutic target.

## 2 MATERIALS AND METHODS

### 2.1 Ethics statement

This study is reported in accordance with ARRIVE guidelines (https://arriveguidelines.org). All procedures involving animals were approved by the Institutional Animal Care and Use Committee (China Academy of Chinese Medical Sciences, Beijing, China). The animal study was carried out in strict accordance with the recommendations in the Guide for the Care and Use of Laboratory Animals of the China Academy of Chinese Medical Sciences (Beijing, China).

### 2.2 Virus

The virus strain used in the study was A/Puerto Rico/8/34 (PR8, H1N1) (ATCC, USA), which is a well‐characterized, mouse‐adapted laboratory strain of influenza A virus. It is used as the genetic backbone for viruses from which inactivated influenza virus vaccines are generated. All experiments with live influenza viruses were performed in an animal biosafety level‐2 (ABSL‐2) laboratory or a biosafety level‐2 (BSL‐2) laboratory.

### 2.3 Base complementation analysis

Since miRNAs are negatively correlated with the expression of their target genes, the expression of viral NP in the host increases after influenza A virus infection; so, we screened miRNAs that were significantly reduced as the target of further research. Our previous studies showed that a 14 miRNAs cluster (Table 1) was the only significantly downregulated miRNA gene cluster in the lung tissue of mice with pneumonia caused by influenza A virus.^16^ Here, miRanda^17^, ^18^ was used to analyse the base complementarity between the *NP* gene (Gene ID: 956531) and the 14 miRNAs mentioned above.

**TABLE 1:** Significantly downregulated miRNA cluster in the lung tissue of mice with pneumonia caused by influenza A virus

miRNA	Accession	Sequence (5′—3′)
mmu‐miR‐30c‐1‐3p	MIMAT0004616	CUGGGAGAGGGUUGUUUACUCC
mmu‐miR‐34b‐3p	MIMAT0004581	AAUCACUAACUCCACUGCCAUC
mmu‐miR‐92b‐3p	MIMAT0004899	UAUUGCACUCGUCCCGGCCUCC
mmu‐miR‐149‐5p	MIMAT0000159	UCUGGCUCCGUGUCUUCACUCCC
mmu‐miR‐375‐3p	MIMAT0000739	UUUGUUCGUUCGGCUCGCGUGA
mmu‐miR‐34c‐3p	MIMAT0004580	AAUCACUAACCACACAGCCAGG
mmu‐miR‐449a‐5p	MIMAT0001542	UGGCAGUGUAUUGUUAGCUGGU
mmu‐miR‐449c‐5p	MIMAT0003460	AGGCAGUGCAUUGCUAGCUGG
mmu‐miR‐411‐3p	MIMAT0001093	UAUGUAACACGGUCCACUAACC
mmu‐miR‐431‐5p	MIMAT0001418	UGUCUUGCAGGCCGUCAUGCA
mmu‐miR‐744‐3p	MIMAT0004820	CUGUUGCCACUAACCUCAACCU
mmu‐miR‐205‐5p	MIMAT0000238	UCCUUCAUUCCACCGGAGUCUG
mmu‐miR‐208a‐5p	MIMAT0017014	GAGCUUUUGGCCCGGGUUAUAC
mmu‐miR‐299a‐3p	MIMAT0004577	UAUGUGGGACGGUAAACCGCUU

The miRanda base complementation analysis follows four rules: (1) bases 2–4 of miRNA must match exactly with the mRNA; (2) mismatches of bases 3–12 of miRNA are ≤5; (3) there is at least one mismatch in the bases 9–25 and (4) the last five bases of miRNA cannot have more than two mismatches. miRanda uses an algorithm similar to that of Smith‐Waterman to construct a scoring matrix that allows G‐U mismatch screening. The complementary scoring rules are as follows: A‐U and G‐C are +5, G‐U is +2, other mismatches are −3, gap penalty is −8, and the gap extension penalty is −2. In order to reflect the heterogeneity of miRNA 5′ end and 3′ end in the process of target gene binding, miRanda software sets the scale parameter, that is, the complementary score of the first 11 bases of the miRNA 5′ end is multiplied by the scale parameter, and the result add the complementary score of 11 bases of the miRNA 3′ end. The result is the final base complementation score of the sequence. Secondly, miRanda utilizes RNAlib from the Vienna software package to calculate the free energy of miRNA binding to the target gene mRNA to estimate the thermodynamic stability of miRNA‐target gene dimerization. Finally, miRanda requires target conservation among multiple species, that is, the target has the same base at the same position in the sequence alignment of multiple species.

### 2.4 Dual luciferase reporter gene analysis

The *NP* gene sequence (5′‐ggcatctgcgggccaaatcagcatacaacctacgttctcagtacagagaaatctcccttttgacagaacaaccgttaTGGCAgCAtt cactgggaatacagaggggagaacatctgacatgaggaccgaaatcataaggatgatggaaagTGCAAGACcagaagatgtgtctttccaggggcggggagtcttcgagctctcggacgaaaaggcagcgagcccgatcgtgccttcctttgacatgagtAATGAAGGAtcttatttcttcggagacaatgcagaggagtacgacaat‐3′) containing miR‐205‐5p, miR‐431‐5p and miR‐744‐3p binding sites was loaded into the pmirGlo vector. MiR‐205‐5p, miR‐431‐5p, miR‐744‐3p mimics and negative control (NC) were synthesized by Genepharma. The 293 T cells were co‐transfected with *NP* gene sequence (using pmirGlo vector) and miR‐205‐5p, miR‐431‐5p, miR‐744‐3p mimics or the NC (using lipofectamine 2000 (Invitrogen)). 293 T cells transfected with pmirGlo vector containing *NP* gene sequence was used as control. The cells were harvested 24 h after co‐transfection, and the luciferase activity was measured with a dual luciferase reporter assay system (Promega E1960) on a multi‐label microplate detector (PerkinElmer EnSpire).^19^, ^20^

### 2.5 MicroRNA functional verification—inhibit the expression of NP synthesized in vitro

The *NP* gene (1525 bp) was loaded into the pcDNA3.1 vector. miR‐205‐5p, miR‐431‐5p, miR‐744‐3p and NC were synthesized by Genepharma. The 293 T cells were co‐transfected with the pcDNA3.1 vector containing *NP* gene and miR‐205‐5p, miR‐431‐5p, miR‐744‐3p or NC (using lipofectamine 2000). Untreated cells served as blank control, those treated with lipofectamine 2000 were the mock control and those transfected with only pcDNA3.1 vector were the vector control.^21^, ^22^ The cells were harvested 48 h after co‐transfection, and Western blot was carried out using the primary antibodies against NP (Abcam) and glyceraldehyde 3‐phosphate dehydrogenase (GAPDH) (Cell Signalling) as an internal control.

### 2.6 MicroRNA functional verification—inhibit the expression of NP from influenza A virus

The mouse lung epithelial (MLE‐12) cell lines were purchased from the BeNa Culture Collection. Cells were cultured in Dulbecco's Modified Eagle Medium (DMEM) containing 10% (v/v) fetal bovine serum (FBS), in a humidified atmosphere of 95% air‐ 5% CO_2_ at 37°C. MLE‐12 cells were cultured in 6 well culture plate, and then the cells were transfected with miR‐205‐5p mimic, miR‐205‐5p inhibitor, miR‐431‐5p mimic, miR‐431‐5p inhibitor, NC or inhibitor negative control (INC), with lipofectamine 2000 for 48 h. miR‐205‐5p inhibitor, miR‐431‐5p inhibitor, INC were synthesized by Genepharma. These miRNA mimic (or respective inhibitor) transfected MLE‐12 cells were simultaneously infected with influenza A virus (MLE‐12 Infection model). The cells cultured in complete medium were considered as normal control. The culture plate was cultured in a 37°C, 5% CO_2_ incubator. The cells were harvested 48 h after transfection, and Western blot was carried out using the primary antibodies against NP (Abcam) and GAPDH (Cell Signalling) as an internal control.^23^

### 2.7 Construction of influenza A virus pneumonia mouse model and drug treatment

Male and female ICR mice (SPF, 14 ± 1 g) were anaesthetized with isoflurane and intranasally administered with 15 times the lethal dose 50% (15 × LD_50_) of PR8 virus suspended in physiological saline. In addition, an uninfected group was administered intranasal physiological saline.^24^ On the day of infection, 27.5 mg/kg/d oseltamivir and 0.88 g/kg/d Jinchai Antiviral Capsules (JC, an anti‐influenza Chinese medicine^25^) were given for four consecutive days. On the 5th day of the virus infection, the mice were sacrificed by cervical dislocation, and lung tissues were dissected. The doses administered to mice are converted from clinical doses.

### 2.8 Dose determination of oseltamivir and JC in MLE‐12 cell experiments

Oseltamivir and JC were prepared into 5 and 50 mg/ml stock solutions with purified water, respectively, and then filtered with sterile membrane (Sartorius). Using the maintenance solution (DMED containing 2% (v/v) FBS) to dilute the stock solution, 2‐fold dilution to eight concentrations, and add to the 96‐well culture plate where MLE‐12 cells have grown into a monolayer (100 μl/well, four duplicate wells for each dilution). At the same time, a normal cell control was set. The culture plate was cultured in a 37°C, 5% CO_2_ incubator, and the cytopathic conditions were observed under an inverted microscope every 24 h for 72 h. Determine the minimum dilution factor at which the cells do not have obvious lesions, calculate the maximum non‐toxic concentration (TC_0_), and calculate the 50% cytotoxic concentration (TC_50_) according to the Reed‐Muench method.^26^ Cytopathy is judged according to six grades (Table 2).

**TABLE 2:** Cytopathic grade 6 criteria

Grade	Cytopathic condition
‐	Cells grow normally, no lesions appear
±	Cytopathic < 10% of the entire monolayer
+	Cytopathies account for <25% of the entire monolayer
++	Cytopathy accounts for <50% of the entire monolayer
+++	Cytopathy accounts for <75% of the entire monolayer
++++	Cytopathies account for >75% of the entire monolayer

A 96‐well culture plate with MLE12 cells grown into a monolayer was taken, and the cell surface was washed three times with cell maintenance solution, then infected with 100 times 50% tissue culture infective dose (100 × TCID_50_) of PR8 virus, and placed in a 37°C, 5% CO_2_ incubator. After the virus was adsorbed for 1 h, the virus solution was discarded, and six dilutions of the drug solution (100 μl/well) below the TC_0_ were sequentially added, and a normal cell control and a virus control were set at the same time. The cells were cultured in a 37°C, 5% CO_2_ incubator, and the cytopathic conditions were observed under an inverted microscope every 24 h. After 72 h, the test results were recorded. The 50% inhibitory concentration (IC_50_) was calculated according to Reed‐Muench method, and the therapeutic index (TI) was calculated, TI = TC_50_/IC_50_.^24^ Cytopathy is judged according to six grades (Table 2).

### 2.9 Cultured MLE‐12 cell infection model and drug treatment

MLE‐12 cells were cultured in culture medium (DMEM containing 10% (v/v) FBS), in a 37°C, 5% CO_2_ incubator. These cultured MLE‐12 cells were infected for 1 h with 100 × TCID_50_ PR8 virus. Discard the virus solution 1 h after infection, and 20 μg/ml oseltamivir and 200 μg/ml JC were given for 48 h. Simultaneously, normal control cells and infected control cells were given equal amount of maintenance solution (DMED containing 2% (v/v) FBS). Oseltamivir and JC were formulated using maintenance solution and filtered through a 70 μm sterile filter (Sartorius).

### 2.10 Quantitative reverse transcriptase‐polymerase chain reaction (qRT‐PCR)

qRT‐PCR was performed to verify the level of *NP*, miR‐205‐5p and miR‐431‐5p in the lung and the MLE‐12 cells. The total RNA extracted (0.5 μg) with Trizol was reverse‐transcribed using M‐MLV reverse transcriptase (Thermo) with random primer for *NP*, and with a special stem‐loop primer (Genepharma) for miR‐205‐5p and miR‐431‐5p. qRT‐PCR was performed on a real‐time PCR system (ABI), using Power SYBR Green PCR Master Mix (ABI). All samples were analysed in fourfold, including the no‐template control. The relative expression level was determined by 2(‐DeltaDeltaC(T)) method and normalized to GAPDH or U6. The primer sequences are shown in Table 3.

**TABLE 3:** Primer sequences in qPCR

Gene	Primer type	Primer sequence
NP	Forward	GTGTATGGACCTGCCGTAGC
	Reverse	TGTGCTGGATTCTCATTTGG
GAPDH	Forward	AACGACCCCTTCATTGACCTC
	Reverse	CCTTGACTGTGCCGTTGAACT
miR‐431‐5p	Forward	ACGCTTGTCTTGCAGGCC
	Reverse	TATGGTTCTTCACGACTGGTTCAC
miR‐205‐5p	Forward	AAGCCGTTCCTTCATTCCAC
	Reverse	TATGGTTGTTCTGCTCTCTGTCTC
U6	Forward	CGCTTCGGCAGCACATATAC
	Reverse	TTCACGAATTTGCGTGTCATC

### 2.11 Western blotting analysis

The protein samples were extracted from the lung tissues or MLE‐12 cells with ExKine™ Total Protein Extraction Kit (abbkine). Protein samples (20 μg) were fractionated using SDS‐PAGE using 10% polyacrylamide gels and then transferred onto a PVDF membrane. After blocking with non‐fat milk, the blots were incubated with the primary antibodies: NP (1:1000, Abcam) and GAPDH (1:2000, Cell Signalling), overnight at 4°C. Subsequently, the secondary antibody incubation was conducted with goat antimouse or goat antirabbit IgG antibody (1:10,000) for 3 h at room temperature. Blots were visualized by ECL (Millipore) and the density of bands was determined by Lane 1D software (Sage).

### 2.12 Histopathologic analysis

The lungs were fixed in 4% formalin, dehydrated in ascending ethanol concentrations, embedded in paraffin, sectioned into 4‐μm‐thick slices, and stained with haematoxylin and eosin (H&E). Histopathology photos were taken using a phase inverted microscope (Olympus).

### 2.13 Statistical analysis

All the results were expressed as mean ± standard deviation (SD). SPSS17.0 software was used for data analysis, analysis of variance (anova) was used to test for homogeneity of variance. Least significant difference (LSD) test was used when the variance was homogeneous; Dunnett T3 test was used when the variance was not homogeneous. Significance level was set at *p* < 0.05; *p* < 0.01 which represents highly significant differences.

## 3 RESULTS

### 3.1 Three MiRNAs, miR‐431‐5p, miR‐744‐3p and miR‐205‐5p, complement the NP gene

**FIGURE 1:**
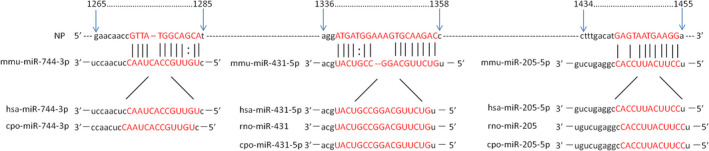
Potential binding site between miR‐744‐5p, miR‐205‐5p or imR‐431‐5p and the NP gene. The complementary nucleotides between miR‐744‐5p, miR‐205‐5p or miR‐431‐5p and the target region of NP gene are connected with short vertical lines.

Using miRanda we predicted that miR‐431‐5p, miR‐744‐3p and miR‐205‐5p were complementary to the *NP* gene, and using mirbase database, we found that the human miR‐431‐5p, miR‐744‐3p and miR‐205‐5p sequence is completely consistent with the mouse sequence, so the results of this study have direct clinical guiding value (Table 4, Figure 1).

**TABLE 4:** MiRNAs complementing the NP gene

miRNA	Gene symbol	Align score	Energy	miRNA start	miRNA end	Gene start	Gene end
mmu‐miR‐205‐5p	NC_002019.1	149	−16.23	2	14	1434	1455
mmu‐miR‐431‐5p	NC_002019.1	157	−24.56	2	19	1336	1358
mmu‐miR‐744‐3p	NC_002019.1	140	−13.11	2	15	1265	1285

### 3.2 miR‐431‐5p and miR‐205‐5p are capable of targeting NP gene

**FIGURE 2:**
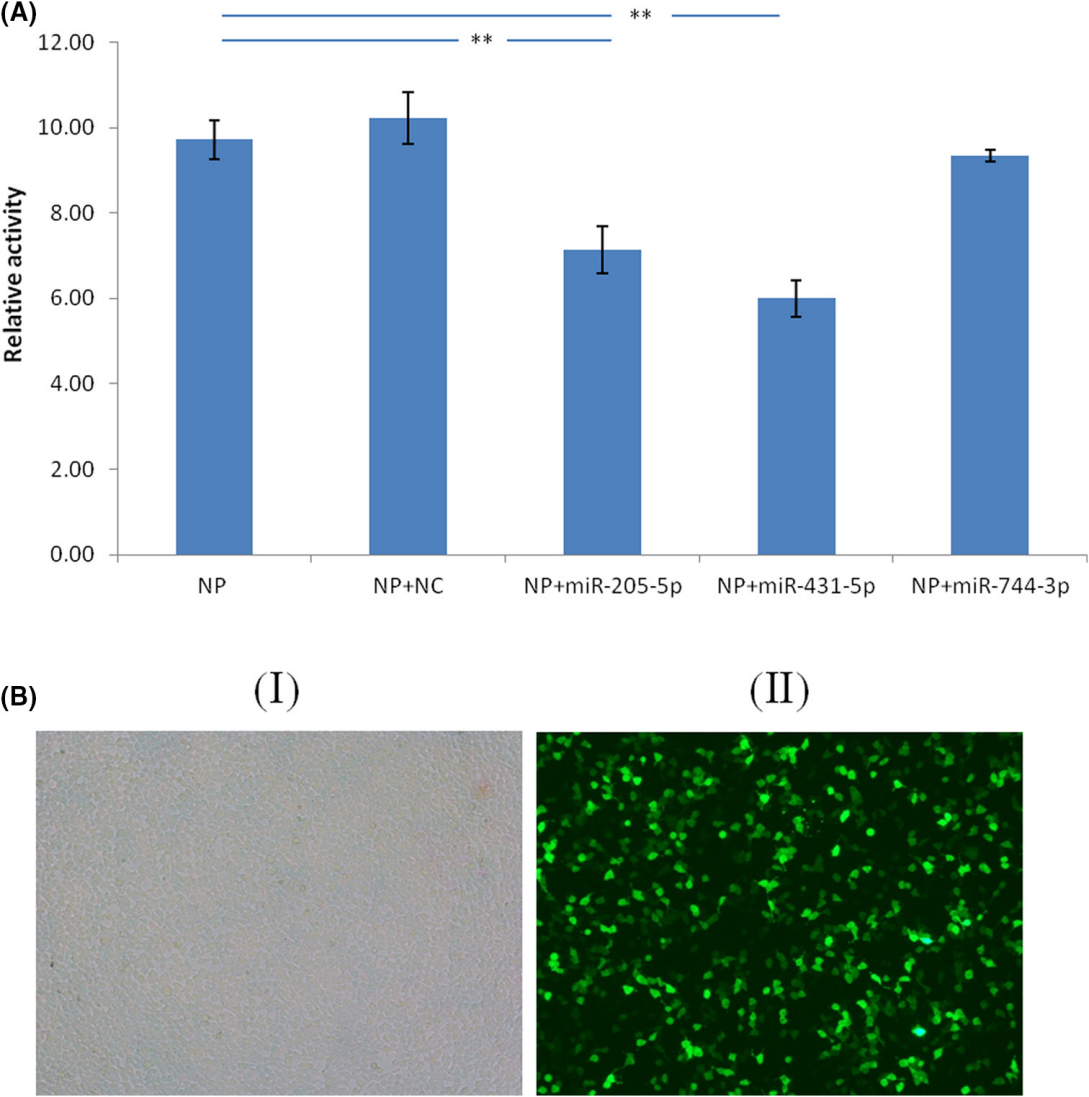
NP is a target of imR‐431‐5p and miR‐205‐5p. (A). Dual luciferase activity assay was performed by co‐transfected pmirGlo vector containing the NP gene sequence with miR‐205‐5p, miR‐431‐5p or miR‐744‐3p into 293 T cells. The dual luciferase activity assay was performed 24 h later. (B) Transfection efficiency of 293 T cells after 24 h (100×); (I) Light microscope of 293 T cells transfected with LV5 labelled negative control miRNA after 24 h, (II) Fluorescence microscope of 293 T cells transfected with LV5 labelled negative control miRNA after 24 h. *n* = 5, **p < 0.01. LV5, a fluorescent marker for labeling miRNA; NC, negative control miRNA

Dual luciferase reporter gene analysis was used to verify the binding of miR‐431‐5p, miR‐744‐3p, or miR‐205‐5p to *NP* gene. Compared with the control, the miR‐205‐5p and miR‐431‐5p mimics strongly suppressed the relative activity of the dual luciferase reporter fused with *NP* gene sequence (Figure 2). The inhibitory effect of miR‐205‐5p and miR‐431‐5p on downstream luciferase gene expression confirmed that miR‐205‐5p and miR‐431‐5p could target the binding site of *NP* gene.

### 3.3 miR‐431‐5p and miR‐205‐5p inhibit the expression of NP synthesized in vitro

**FIGURE 3:**
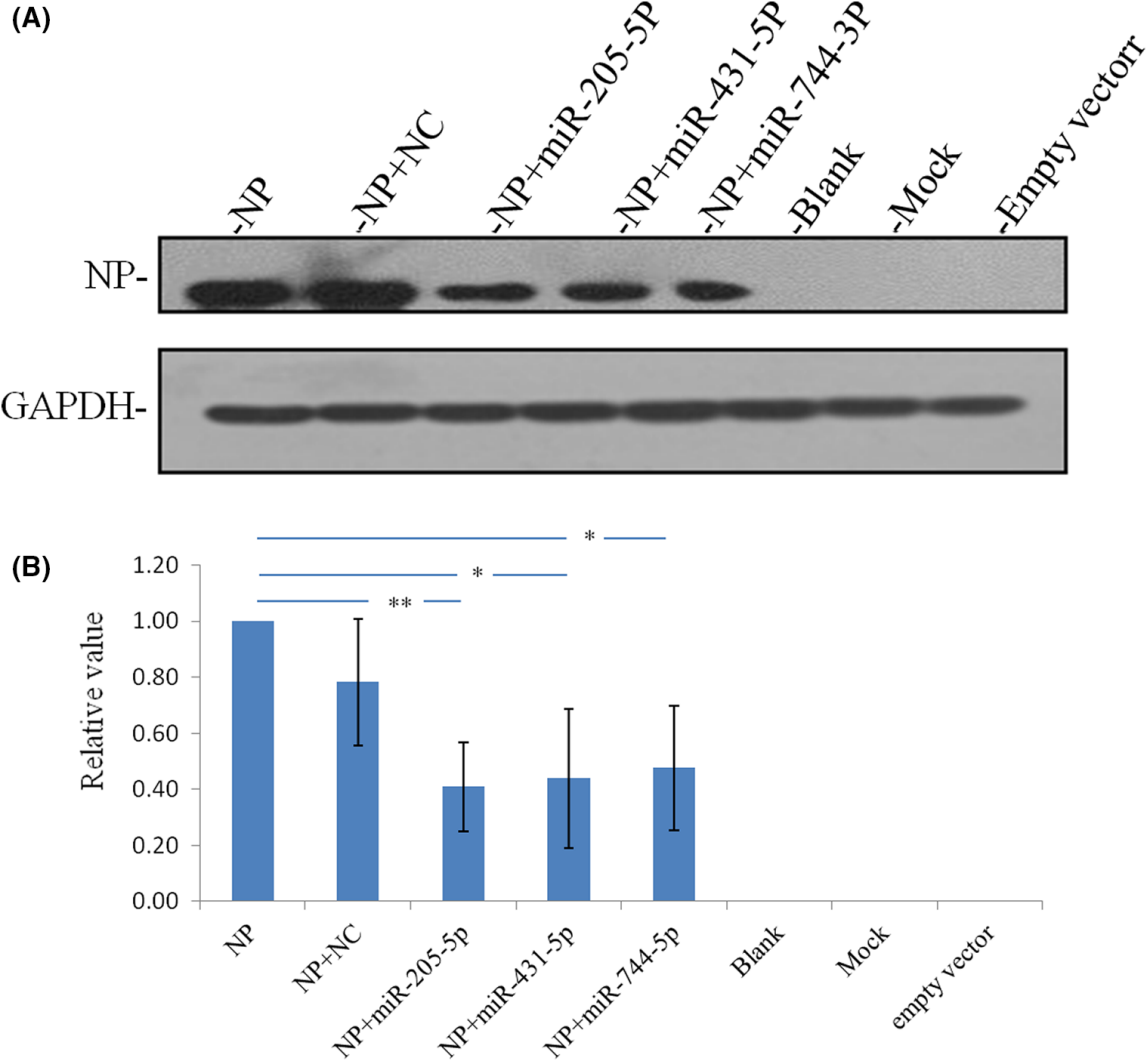
NP protein expression is highly significantly inhibited by miR‐205‐5p and is significantly inhibited by miR‐431‐5p and miR‐744‐3p. *n* = 3; highly significant **p < 0.01; significant *p < 0.05

The *NP* whole gene eukaryotic cell overexpression analysis was used to further verify the inhibitory effect of miR‐205‐5p, miR‐744‐3p or miR‐431‐5p on *NP* expression via *NP* gene binding. Western blotting was performed to examine the NP expression level. Compared with that of the control, miR‐205‐5p, miR‐431‐5p and miR‐744‐3p all can inhibit NP expression levels (Figure 3). However, the dual luciferase assay showed that miR‐744‐3p could not bind its specific site to inhibit downstream gene expression, so there may be other pathways for the inhibition of NP expression by miR‐744‐3p, the reasons for which will be discussed in detail later.

### 3.4 miR‐205‐5p inhibit the expression of NP from influenza A virus

**FIGURE 4:**
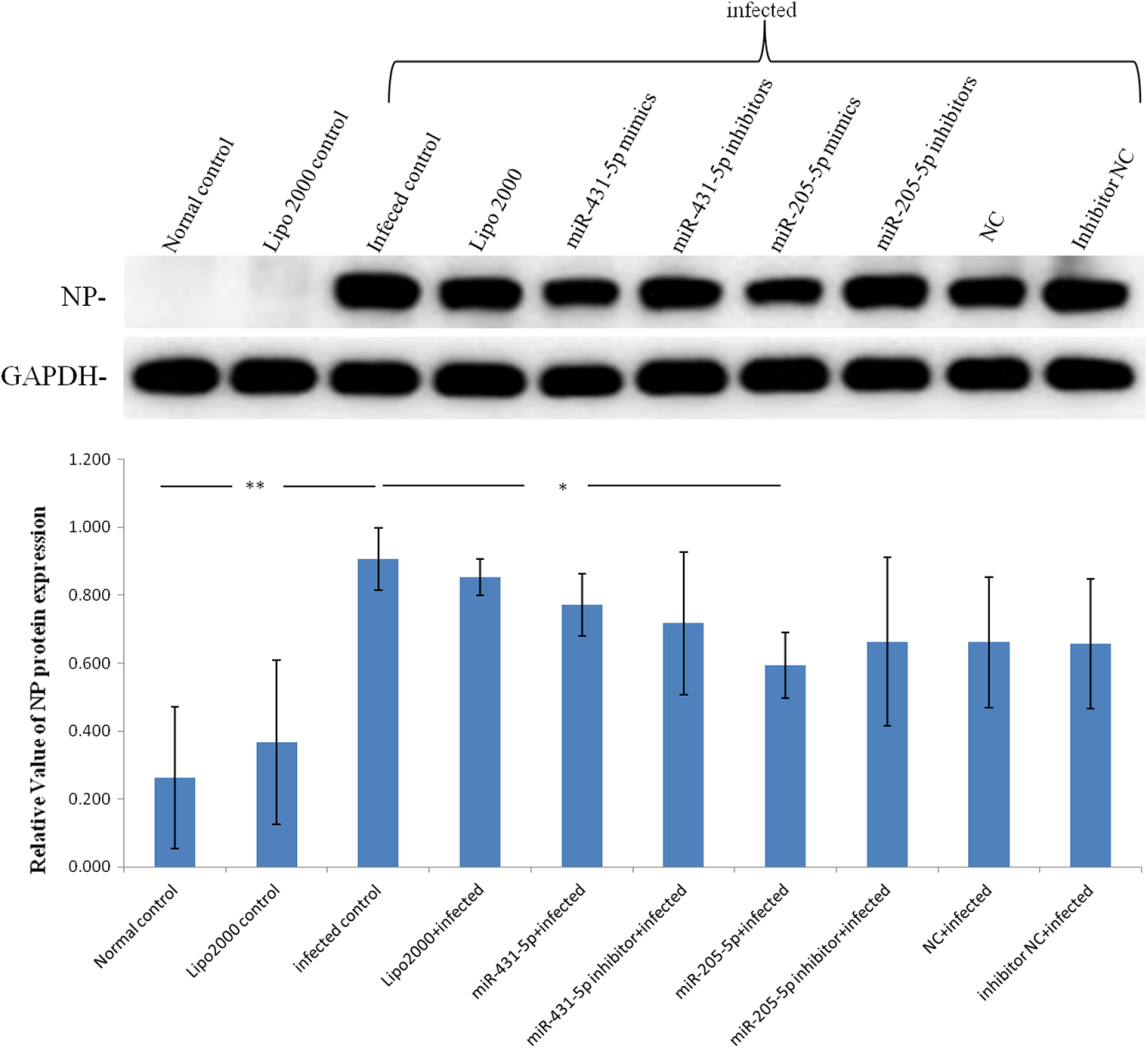
MiR‐205‐5p inhibited NP protein expression in influenza A virus MLE‐12 cells. The level of NP protein was highly significantly up‐regulated in infected group, but significantly down‐regulated in miR‐205‐5p + infected group. *n* = 4; highly significant **p < 0.01; significant *p < 0.05

MLE‐12 cells over‐expressing miR‐205‐5p or miR‐431‐5p were used to verify their inhibition on influenza A virus replication. Western blotting assay was carried out to check NP expression levels. NP expression was significantly decreased in influenza A virus infected MLE‐12 cells overexpressing miR‐205‐5p (Figure 4). Therefore, in host cells, up‐regulation of miR‐205‐5p levels is important for suppressing influenza A virus NP expression. Further, NP expression level was used to evaluate the influenza A virus replication ability, so up‐regulation of host miR‐205‐5p expression can inhibit influenza A virus replication. In conclusion, miR‐205‐5p can be used as a therapeutic target for influenza A.

### 3.5 miR‐205‐5p: A potential target for drugs used to treat influenza A viral infection

**FIGURE 5:**
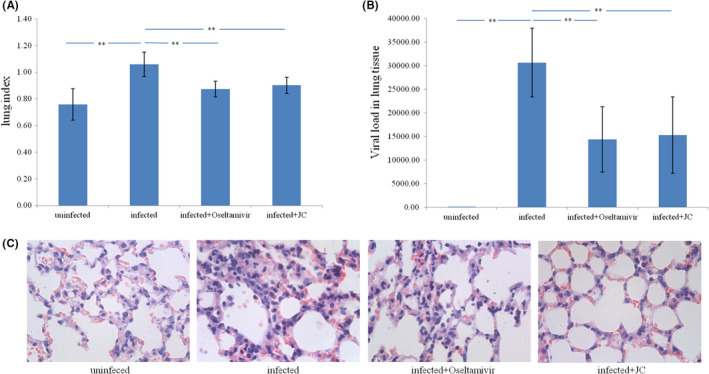
A mouse model of influenza A virus pneumonia and drugs treatment. (A) Influenza A virus infection causes a significant increase in the lung index, which could be significantly reduced by oseltamivir and JC. Lung index = wet lung weight (g)/body weight (g) × 100. (B) Influenza A virus infection causes a significant increase in the viral load of lung tissue, which could be significantly reduced by oseltamivir and JC. (C) In the uninfected group, there was no inflammatory infiltration in the lung tissue, and the structure was normal; In the infection group, there were a large number of cell proliferation, inflammatory cell exudation and exfoliated cells in the lung tissue, and the alveolar cavity size varies; In the Oseltamivir and JC group, inflammation and edema in the lung tissues were reduced. (H&E, 400×). (*n* = 10; **p < 0.01)

We created a mouse pneumonia model using influenza A virus and treated with oseltamivir and JC. Influenza A virus infection causes a highly significant lung index increase. This lung index increase could be significantly reduced by oseltamivir and JC (Figure 5A). Detection of *NP* expression levels indicative of viral load in lung tissue. Influenza A virus infection causes a highly significant viral load increase. This viral load increase could be significantly reduced by oseltamivir and JC (Figure 5B). Influenza A virus infection caused several pathological changes: increased cell proliferation, inflammatory cell exudation, cell exfoliation and varied alveolar cavity size were observed in the lung tissue. These changes could be improved by oseltamivir and JC (Figure 5C). The above data show that oseltamivir and JC have a significant effect on influenza A virus pneumonia.

**FIGURE 6:**
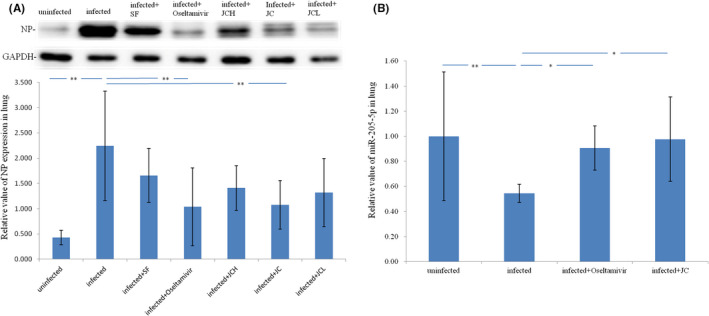
In vivo the effect of oseltamivir and JC on the expression of host miR‐205‐5p was negatively correlated with the effect on the expression of viral NP. (A) NP protein up‐regulate in influenza A virus infected lung tissue and down‐regulated by Oseltamivir and JC; *n* = 6; **p < 0.01; JCH, twice the clinical dose of JC; JCL, half the clinical dose of JC; SF, Shufeng Jiedu capsule, an anti‐influenza Chinese medicine. (B) miR‐205‐5p down‐regulate in influenza A virus infected lung tissue and up‐regulated by Oseltamivir and JC; *n* = 8; **p < 0.01; *p < 0.05

Differential expression of NP protein in influenza A virus infected lung tissue were verified by Western blot analysis. NP protein expression level was confirmed to be up‐regulated in influenza A virus infected lung tissue and be down‐regulated by oseltamivir and JC (Figure 6A). Differential expression of miR‐205‐5p in influenza A virus infected lung tissue were verified by qRT‐PCR. miR‐205‐5p expression was confirmed to be significantly down‐regulated in influenza A virus infected lung tissue and could be up‐regulated by oseltamivir and JC (Figure 6B). The above results showed that the effects of oseltamivir and JC on host miR‐205‐5p expression were negatively correlated with their effects on viral NP expression in vivo.

The drug cytotoxicity test results show that the maximum non‐toxic concentration can be obtained by diluting oseltamivir and JC original drug solution by 64 times. The original drug concentrations of oseltamivir and JC are 5 and 50 mg/ml respectively, so the TC_0_ is respectively 0.078 and 0.78 mg/ml (Table 5). The TC_50_ of oseltamivir and JC calculated by Reed‐Muench method are 0.112 and 1.12 mg/ml respectively (Table 5). The results of the antiviral experiment of the drug on cells showed that oseltamivir still had a good antiviral effect at the lowest dilution (1024‐fold dilution), while JC had a good antiviral effect at a 256‐fold dilution (Table 6). The IC_50_ of JC calculated by the Reed‐Muench method was 0.12 mg/ml, while the IC_50_ of oseltamivir could not be calculated due to its good efficacy at the lowest dilution (Table 6). Based on the above experimental results, the in vitro experimental doses of oseltamivir and JC were both selected at 256‐fold dilution, that is, 0.02 and 0.2 mg/ml.

**TABLE 5:** Toxicity of oseltamivir and JC on MLE‐12 cells

Group	Dilution ratio	Lesion grade	TC_50_	TC_0_
Cell control	×	0000	×	×
Oseltamivir	1:2	4444	0.112 mg/ml	0.078 mg/ml
	1:4	4444		
	1:8	4444		
	1:16	3333		
	1:32	2222		
	1:64	0000		
	1:128	0000		
	1:256	0000		
JC	1:2	4444	1.120 mg/ml	0.780 mg/ml
	1:4	4444		
	1:8	4444		
	1:16	4444		
	1:32	3333		
	1:64	0000		
	1:128	0000		
	1:256	0000		

*Note*: The original concentration of oseltamivir and JC are 5 mg/ml and 50 mg/ml; cytopathic score: ‘–’ = 0, ‘+’ = 1, ‘++’ = 2, ‘+++’ = 3, ‘++++’ = 4, 4 duplicate holes were scored, respectively.

**TABLE 6:** Antiviral effects of oseltamivir and JC on MLE‐12 cells

Group	Dilution ratio	Lesion grade	IC_50_	TI
Cell control	×	0000	×	×
Viral control	×	4444	×	×
Oseltamivir	1:64	2002	×	×
	1:128	0000		
	1:256	0000		
	1:512	0000		
	1:1024	0000		
JC	1:64	4444	0.12 mg/ml	9.33
	1:128	2000		
	1:256	0000		
	1:512	4004		
	1:1024	4444		

*Note*: The original concentration of oseltamivir and JC are 5 mg/ml and 50 mg/ml; cytopathic score: ‘–’ = 0, ‘+’ = 1, ‘++’ = 2, ‘+++’ = 3, ‘++++’ = 4, 4 duplicate holes were scored, respectively.

**FIGURE 7:**
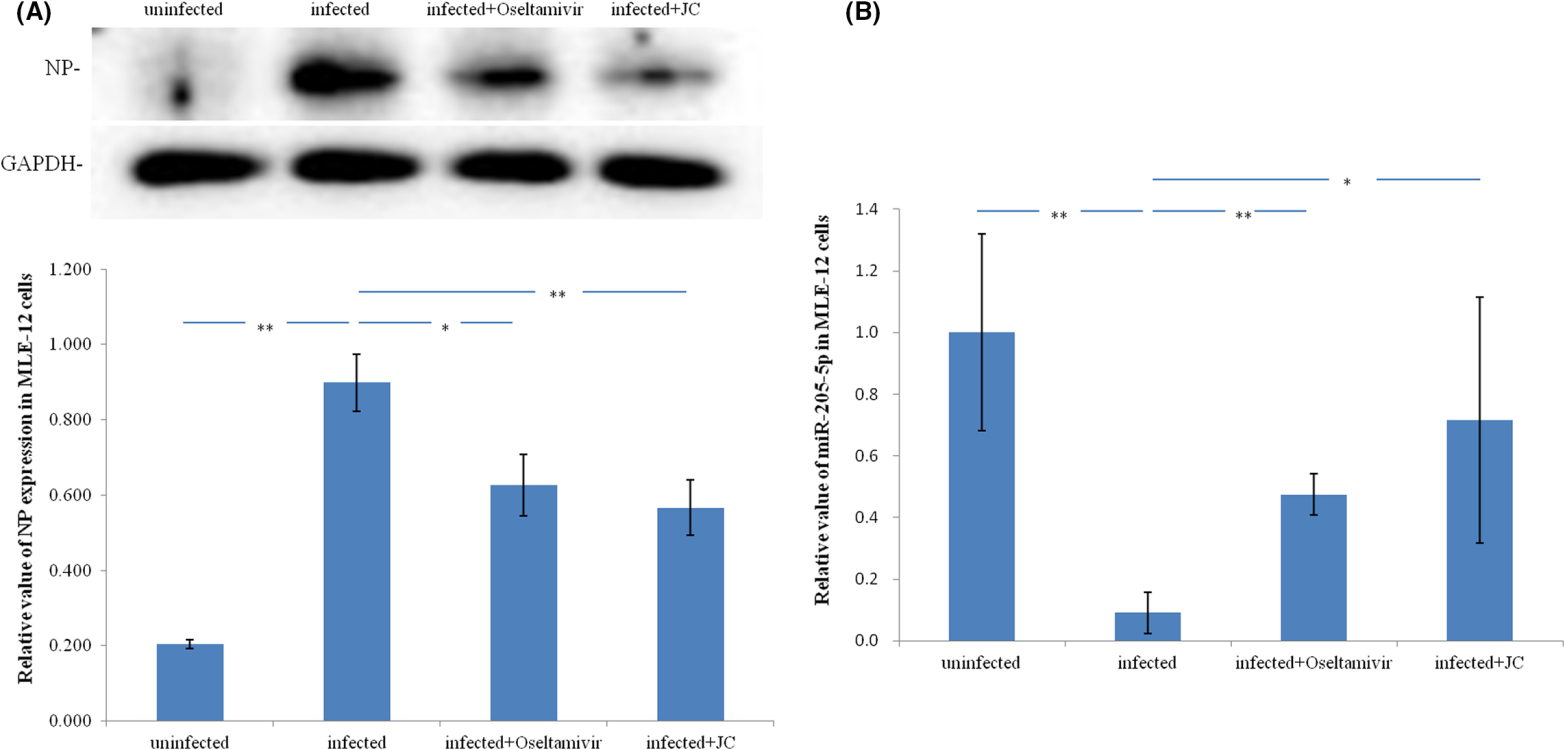
In vitro the effect of oseltamivir and JC on the expression of host mir‐205‐5p was negatively correlated with the effect on the expression of viral NR. (A) NP protein up‐regulate in influenza A virus infected MLE‐12 cells and down‐regulated by Oseltamivir and JC; *n* = 3; **p < 0.01; *p < 0.05. (B) miR‐205‐5p down‐regulate in influenza A virus infected MLE‐12 cells and up‐regulated by Oseltamivir and JC; *n* = 4; **p < 0.01; *p < 0.05

Differential expression of NP protein in influenza A virus infected MLE‐12 cells were verified by Western blot analysis. NP protein expression level was confirmed to be up‐regulated in influenza A virus infected MLE‐12 cells and be down‐regulated by oseltamivir and JC (Figure 7A). Differential expression miR‐205‐5p in influenza A virus infected MLE‐12 cells were verified by qRT‐PCR. miR‐205‐5p expression was confirmed to be significantly downregulated in influenza A virus infected MLE‐12 cells, and could be upregulated by oseltamivir and JC (Figure 7B). The above results showed that the effects of oseltamivir and JC on host miR‐205‐5p expression were also negatively correlated with their effects on viral NP expression in vitro.

In summary, In vitro and in vivo experiments, under the premise of ensuring that oseltamivir and JC have anti‐influenza efficacy, both oseltamivir and JC can down‐regulate the expression level of viral NP protein, while the expression level of miR‐205‐5p was up‐regulated, suggesting that miR‐205‐5p is a therapeutic target of anti‐influenza A drugs.

## 4 DISCUSSION

The World Health Organization has always believed that influenza virus may be one of the most potential threats to humans.^27^ In recent years, with the increase in the scope of global species activities, the probability of influenza virus gene recombination among humans, poultry, pigs and other species has greatly increased, which has enhanced the variability of influenza viruses.^28^ At the same time, due to the wide application of anti‐influenza virus drugs, the drug resistance of influenza virus is becoming more and more serious. Therefore, it is urgent to develop new anti‐influenza drugs and find new therapeutic targets.

The influenza A virus, belonging to the Orthomyxoviridae family, can cause severe lung infections and even death.^29^ The therapeutic targets of influenza A have been mainly viral proteins, such as ion channel protein (M2),^30^, ^31^, ^32^, ^33^, ^34^ neuraminidase (NA),^35^, ^36^, ^37^, ^38^ hemagglutinin (HA),^39^, ^40^ nucleoprotein (NP)^41^ and RNA‐dependent RNA polymerase (RdRp).^42^ At present, most of the influenza A treatment drugs use these proteins as therapeutic targets; these anti‐influenza A drugs will show good antiviral ability in the early stage of use, but due to the drug resistance of the virus, it is necessary to continuously develop new antiviral drugs drug. In recent years, in order to solve the problem of drug resistance of anti‐Influenza A drugs, scholars have been constantly looking for new antiviral targets that are not easily resistant to drug resistance to develop new antiviral drugs, among which host targets are the most studied.^43^, ^44^ In this study, we took host factors as a breakthrough to screen and mine the target of anti‐influenza A virus drugs, and miR‐205‐5p was successfully identified as an antiviral drug target candidate.

MiRNAs participate in many biological host cell processes and are an important biological factor for host biological balance maintenance.^45^, ^46^, ^47^, ^48^ miRNAs degrade target genes or blocks translation through complementary binding to target genes, thereby inhibiting protein expression.^49^ A viral infection represents interplay between two biological systems—the host cell and the virus. when these two biological systems are at interplay, host miRNAs could regulate the replication and pathogenicity of viruses. For example, simian foam virus type 1 (PFV‐1),^13^ vesicular stomatitis virus (VSV),^50^ hepatitis C virus (HCV),^51^, ^52^ and human immunodeficiency virus (HIV‐1)^53^, ^54^ affect the viral infection processes by modulating viral gene expression, While miR‐323, miR‐491 and miR‐654 can target the same conserved region of the influenza A virus *PB1* gene, let‐ 7c can target the *M* gene of influenza A virus, they all can inhibit in vitro replication of influenza A virus.^14^, ^15^ However, miRNA have not been systematically researched as the target for anti‐influenza A viral therapy. We have been working to find novel host targets against influenza A virus, and in recent years have targeted host miRNAs. Earlier, we reported that a cluster of 14 miRNAs—viz. miR‐30c‐1‐3p, miR‐34b‐3p, miR‐92b‐3p, miR‐149‐5p, miR‐375‐3p, miR‐34c‐3p, miR‐449a‐5p, miR‐449c‐5p, miR‐411‐3p, miR‐431‐5p, miR‐744‐3p, miR‐205‐5p, miR‐208a‐5p, and miR‐299a‐3p—is downregulated in the lung tissue of influenza A virus infected pneumonic mice.^16^ In this study, miRanda was used to analyse the base complementarity between the influenza A virus gene and the 14 miRNAs mentioned above. MiR‐431‐5p, miR‐744‐3p, and miR‐205‐5p were predicted to be complementary to the *NP* gene by miRanda and could be further explored as candidate research targets.

The nucleoprotein (NP) is indispensable for the transcription and replication of influenza A virus.^11^ In the viral particles, the NP wraps the viral nucleic acid playing a protective role. The NP antigen structure is stable. Together with the matrix protein, it determines the virus type specificity and rarely undergoes mutation. It forms a ribonucleoprotein complex with RNA polymerases PB2, PB1, and PA to aid viral transcription and replication.^55^ *NP* gene is not only closely associated with influenza A virus replication, but also has a large number of conserved sequences. Therefore, exploring biological mechanisms that can regulate the *NP* gene expression and inhibit viral replication has important research significance. In this study, it is important to confirm the candidate anti‐influenza A virus target (miR‐431‐5p, miR‐744‐3p or miR‐205‐5p) inhibit the expression of NP protein by complementing the *NP* gene, and is valuable for clarifying their status of antiviral target. Dual‐luciferase reporter gene analysis and miRNA functional validation experiments were performed to validate our ideas.

We co‐transfected 293 T cells with *NP* gene sequence containing special sites and miR‐205‐5p, miR‐431‐5p or miR‐744‐3p mimics. Dual luciferase reporter gene assays carried out on these co‐transfected 293 T cells confirmed that miR‐205‐5p and miR‐431‐5p can bind to *NP* gene. Similarly, 293 T cells were co‐transfected with *NP* gene and miR‐205‐5p, miR‐431‐5p or miR‐744‐3p mimics. Western blot assays on these co‐transfected 293 T cells showed that miR‐205‐5p, miR‐431‐5p and miR‐744‐3p can inhibit NP expression. Taken together, these results confirmed that miR‐205‐5p and miR‐431‐5p inhibited NP expression by binding its *NP* gene. Further, functional verification of miR‐205‐5p and miR‐431‐5p mediated inhibition of NP expression was performed using miRNA over‐expression system. MLE‐12 cells were simultaneously infected with influenza A virus and stimulated to over‐express miRNA mimics (miR‐205‐5p or miR‐431‐5p) for 48 h. Western blot was performed to examine NP expression levels. We found that NP expression during influenza A virus infection is significantly reduced in MLE‐12 cells over‐expressing the miR‐205‐5p mimic. These findings suggest that host miRNA‐205‐5p can regulate the expression of influenza A virus NP protein by binding to the *NP* gene, which may be a candidate for drug therapy targets.

The conclusion that miR‐205‐5p can be used as a therapeutic target for anti‐influenza A drugs needs to be confirmed by drug experiments. In this study, we used a mouse model of influenza A virus pneumonia to validate that miRNA‐205‐5p is a therapeutic target for anti‐influenza A drug. Findings showed that pathological changes, including the lung index increase, viral load increase, tissue lesions enhance, and NP protein expression increase caused by influenza A virus infection can be alleviated by oseltamivir (a neuramidase inhibitor which inhibits viral budding) and JC (a traditional chinese medicine which treat influenza A), and miR‐205‐5p expression decrease can be enhanced by oseltamivir and JC. The results suggest that miR‐205‐5p can be used as a therapeutic target for anti‐influenza A drugs. The results suggest that miR‐205‐5p can be used as a therapeutic target for anti‐influenza A drugs. Only two typical drugs were validated in this study, and there are many more drugs to be validated in the future.

## 5 CONCLUSION

We provide new evidence that miR‐205‐5p plays an important regulation role during influenza A virus infection. The regulation effect of miR‐205‐5p on influenza A virus replication could be attributed to the inhibition of NP expression. Simultaneously, we confirmed that the expression of miR‐205‐5p in vivo and in vitro can be up‐regulated by oseltamivir and JC, suggesting that miR‐205‐5p is the target of oseltamivir and JC against influenza A virus. Moreover, here, we have studied effects of miRNA on segments of a single influenza A virus gene. Our future research will verify these findings for other gene segments. This study represents a major effort to elucidate antiviral molecular mechanisms dependent on host miRNAs to target influenza A virus infections. We provide a potential drug target—miR‐205‐5p. The results of this study provide new research ideas and approaches for the future research and development of new anti‐influenza A virus drugs target and the in‐depth study of antiviral mechanisms.

## AUTHOR CONTRIBUTIONS

**yanyan bao:** Conceptualization (lead); data curation (lead); formal analysis (lead); funding acquisition (lead); investigation (lead); methodology (lead); project administration (lead). **yujing shi:** Investigation (supporting). **lirun zhou:** Investigation (supporting). **shuangrong gao:** Investigation (supporting). **rongmei yao:** Investigation (supporting). **shanshan guo:** Investigation (supporting). **zihan geng:** Investigation (supporting). **lei bao:** Investigation (supporting). **ronghua zhao:** Investigation (supporting). **xiaolan cui:** Project administration (supporting).

## CONFLICT OF INTEREST

The authors confirm that there are no conflicts of interest.

## Data Availability

All data generated or analysed during this study are included in this published article.

## References

[1] Schweiger B , Zadow I , Heckler R . Antigenic drift and variability of influenza viruses. Med Microbiol Immunol. 2002;191(3–4):133‐138. 12458347 10.1007/s00430-002-0132-3

[2] Taubenberger JK , Kash JC , Morens DM . The 1918 influenza pandemic: 100 years of questions answered and unanswered. Sci Transl Med. 2019;11(502):eaau5485. 31341062 10.1126/scitranslmed.aau5485 PMC11000447

[3] Taubenberger JK , Morens DM . The 1918 influenza pandemic and its legacy. Cold Spring Harb Perspect Med. 2020;10(10):a038695. 31871232 10.1101/cshperspect.a038695 PMC7528857

[4] Lampejo T . Influenza and antiviral resistance: an overview. Eur J Clin Microbiol Infect Dis. 2020;39(7):1201‐1208. 32056049 10.1007/s10096-020-03840-9 PMC7223162

[5] Pizzorno A , Abed Y , Boivin G . Influenza drug resistance. Semin Respir Crit Care Med. 2011;32(4):409‐422. 21858746 10.1055/s-0031-1283281

[6] Kenneth Baillie J , Digard P . Influenza–time to target the host? N Engl J Med. 2013;369(2):191‐193. 23841736 10.1056/NEJMcibr1304414

[7] Mohl G , Liddle N , Nygaard J , et al. Novel influenza inhibitors designed to target PB1 interactions with host importin RanBP5. Antiviral Res. 2019;164:81‐90. 30742842 10.1016/j.antiviral.2019.02.003

[8] Alleva LM , Cai C , Clark IA . Using complementary and alternative medicines to target the host response during severe influenza. Evid Based Complement Alternat Med. 2010;7(4):501‐510. 19779008 10.1093/ecam/nep152 PMC2892358

[9] Albo C , Valencia A , Portela A . Identification of an RNA binding region within the N‐terminal third of the influenza a virus nucleoprotein. J Virol. 1995;69(6):3799‐3806. 7745727 10.1128/jvi.69.6.3799-3806.1995 PMC189097

[10] Boulo S , Akarsu H , Ruigrok RW , et al. Nuclear traffic of influenza virus proteins and ribonucleoprotein complexes. Virus Res. 2007;124(1–2):12‐21. 17081640 10.1016/j.virusres.2006.09.013

[11] Liu M , Lam MK , Zhang Q , Elderfield R , Barclay WS , Shaw PC . The functional study of the N‐terminal region of influenza B virus nucleoprotein. PLoS One. 2015;10(9):e0137802. 26368391 10.1371/journal.pone.0137802 PMC4569402

[12] Cuellar TL , McManus MT . MicroRNAs and endocrine biology. J Endocrinol. 2005;187:327‐332. 16423811 10.1677/joe.1.06426

[13] Lecellier CH , Dunoyer P , Arar K , et al. A cellular microRNA mediates antiviral defense in human cells. Science. 2005;308(5721):557‐560. 15845854 10.1126/science.1108784

[14] Song L , Liu H , Gao S , Jiang W , Huang W . Cellular microRNAs inhibit replication of the H1N1 influenza a virus in infected cells. J Virol. 2010;84(17):8849‐8860. 20554777 10.1128/JVI.00456-10 PMC2919005

[15] Ma YJ , Yang J , Fan XL , et al. Cellular microRNA let‐7c inhibits M1 protein expression of the H1N1 influenza a virus in infected human lung epithelial cells. J Cell Mol Med. 2012;16(10):2539‐2546. 22452878 10.1111/j.1582-4934.2012.01572.x PMC3823446

[16] Bao Y , Gao Y , Jin Y , Cong W , Pan X , Cui X . MicroRNA expression profiles and networks in mouse lung infected with H1N1 influenza virus. Mol Genet Genomics. 2015;290(5):1885‐1897. 25893419 10.1007/s00438-015-1047-1

[17] Baruah V , Bose S . Computational identification of hepatitis E virus‐encoded microRNAs and their targets in human. J Med Virol. 2019;91(8):1545‐1552. 30919453 10.1002/jmv.25471

[18] Zhang X , Li C , Zhang B , et al. Differential expression and correlation analysis of miRNA‐mRNA profiles in swine testicular cells infected with porcine epidemic diarrhea virus. Sci Rep. 2021;11(1):1868. 33479333 10.1038/s41598-021-81189-5 PMC7820490

[19] Wang Y , Zheng ZJ , Jia YJ , Yang YL , Xue YM . Role of p53/miR‐155‐5p/sirt1 loop in renal tubular injury of diabetic kidney disease. J Transl Med. 2018;16(1):146. 29848325 10.1186/s12967-018-1486-7 PMC5975703

[20] Sun L , Lian JX , Meng S . MiR‐125a‐5p promotes osteoclastogenesis by targeting TNFRSF1B. Cell Mol Biol Lett. 2019;24:23. 30976285 10.1186/s11658-019-0146-0 PMC6437974

[21] Tanhaeian A , Azghandi M , Mousavi Z , Javadmanesh A . Expression of thanatin in HEK293 cells and investigation of its antibacterial effects on some human pathogens. Protein Pept Lett. 2020;27(1):41‐47. 31438823 10.2174/0929866526666190822162140 PMC6978649

[22] Zhang WT , Tang J , Zhao HM , You JY . Construction of rat interleukin‐10 adenoviral vector and its expression in bone marrow mesenchymal stem cells. Zhongguo Dang Dai Er Ke Za Zhi. 2019;21(7):708‐712. 31315773 10.7499/j.issn.1008-8830.2019.07.017 PMC7389103

[23] Bao Y , Lin C , Ren J , Liu J . MicroRNA‐384‐5p regulates ischemia‐induced cardioprotection by targeting phosphatidylinositol‐4,5‐bisphosphate 3‐kinase, catalytic subunit delta (PI3K p110δ). Apoptosis. 2013;18(3):260‐270. 23315007 10.1007/s10495-013-0802-1

[24] Guo S , Bao L , Li C , Sun J , Zhao R , Cui X . Antiviral activity of iridoid glycosides extracted from fructus Gardeniae against influenza a virus by PACT‐dependent suppression of viral RNA replication. Sci Rep. 2020;10(1):1897. 32024921 10.1038/s41598-020-58443-3 PMC7002373

[25] Zhong J , Cui X , Shi Y , Gao Y , Cao H . Antiviral activity of Jinchai capsule against influenza virus. J Tradit Chin Med. 2013;33(2):200‐204. 23789217 10.1016/S0254-6272(13)60125-9 PMC7147227

[26] Reed LJ , Hugo M . A simple method for estimating fifty percent endpoints. Am J Hyg. 1938;27:493‐497.

[27] Abdel‐Ghafar AN , Chotpitayasunondh T , Gao Z , et al. Update on avian influenza a (H5N1) virus infection in humans. N Engl J Med. 2008;358(3):261‐273. 18199865 10.1056/NEJMra0707279

[28] Herfst S , Imai M , Kawaoka Y , et al. Avian influenza virus transmission to mammals. Curr Top Microbiol Immunol. 2014;385:137‐155. 25048542 10.1007/82_2014_387

[29] Nelson MI , Holmes EC . The evolution of epidemic influenza. Nat Rev Genet. 2007;8(3):196‐205. 17262054 10.1038/nrg2053

[30] Trist IM , Nannetti G , Tintori C , et al. 4, 6‐Diphenylpyridines as promising novel anti‐influenza agents targeting the PA‐PB1 protein‐protein interaction: structure‐activity relationships exploration with the aid of molecular modeling. J Med Chem. 2016;59(6):2688‐2703. 26924568 10.1021/acs.jmedchem.5b01935

[31] Wang Y , Hu Y , Xu S , et al. In vitro pharmacokinetic optimizations of AM2‐S31N channel blockers led to the discovery of slow‐binding inhibitors with potent antiviral activity against drug‐resistant influenza a viruses. J Med Chem. 2018;61(3):1074‐1085. 29341607 10.1021/acs.jmedchem.7b01536 PMC6445276

[32] Wu Y , Canturk B , Jo H , et al. Flipping in the pore: discovery of dual inhibitors that bind in different orientations to the wild‐type versus the amantadine‐resistant S31N mutant of the influenza a virus M2 proton channel. J Am Chem Soc. 2014;136(52):17987‐17995. 25470189 10.1021/ja508461m PMC4286326

[33] Hu Y , Musharrafieh R , Ma C , et al. An M2‐V27A channel blocker demonstrates potent in vitro and in vivo antiviral activities against amantadine‐sensitive and‐resistant influenza a viruses. Antiviral Res. 2017;140:45‐54. 28087313 10.1016/j.antiviral.2017.01.006 PMC5326599

[34] Musharrafieh R , Ma C , Wang J . Discovery of M2 channel blockers targeting the drug‐resistant double mutants M2‐S31N/L26I and M2‐S31N/V27A from the influenza a viruses. Eur J Pharm Sci. 2020;141:105124. 31669761 10.1016/j.ejps.2019.105124 PMC6951800

[35] Wu X , Wu X , Sun Q , et al. Progress of small molecular inhibitors in the development of anti‐influenza virus agents. Theranostics. 2017;7(4):826‐845. 28382157 10.7150/thno.17071 PMC5381247

[36] Leang SK , Kwok S , Sullivan SG , et al. Peramivir and laninamivir susceptibility of circulating influenza a and B viruses. Influenza Other Respi Viruses. 2014;8(2):135‐139. 10.1111/irv.12187 PMC4186459 24734292

[37] Kakuta M , Kubo S , Tanaka M , et al. Efficacy of a single intravenous administration of laninamivir (an active metabolite of laninamivir octanoate) in an influenza virus infection mouse model. Antiviral Res. 2013;100(1):190‐195. 23954190 10.1016/j.antiviral.2013.08.004

[38] Ison MG . Clinical use of approved influenza antivirals: therapy and prophylaxis. Influenza Other Respi Viruses. 2013;7(Suppl 1):7‐13. 10.1111/irv.12046 PMC5978625 23279892

[39] Zeng LY , Yang J , Liu S . Investigational hemagglutinin‐targeted influenza virus inhibitors. Expert Opin Investig Drugs. 2017;26(1):63‐73. 10.1080/13543784.2017.1269170 27918208

[40] Kadamr U , Wilsoni A . Structural basis of influenza virus fusion inhibition by the antiviral drug Arbidol. Proc Natl Acad Sci U S A. 2017;114(2):206‐214. 28003465 10.1073/pnas.1617020114 PMC5240704

[41] Lejal N , Tarus B , Bouguyon E , et al. Structure‐based discovery of the novel antiviral properties of naproxen against the nucleoprotein of influenza a virus. Antimicrob Agents Chemother. 2013;57(5):2231‐2242. 23459490 10.1128/AAC.02335-12 PMC3632891

[42] Buck KW , Comparison of the replication of positivestranded rna viruses of plants and animal. 1996:159–251. 10.1016/S0065-3527(08)60736-8 PMC7131377 8895833

[43] O'Hanlon R , Leyva‐Grado VH , Sourisseau M , et al. An influenza virus entry inhibitor targets class II pi3 kinase and synergizes with oseltamivir. ACS Infect Dis. 2019;5(10):1779‐1793. 31448902 10.1021/acsinfecdis.9b00230

[44] Li C , Xu LJ , Lian WW , et al. Anti‐influenza effect and action mechanisms of the chemical consti‐tuent gallocatechin‐7‐gallate from *Pithecellobium clypearia* Benth. Acta Pharmacol Sin. 2018;39(12):1913‐1922. 29802302 10.1038/s41401-018-0030-x PMC6289332

[45] Hwang HW , Mendell JT . MicroRNAs in cell proliferation, cell death, and tumorigenesis. Br J Cancer. 2006;94(6):776‐780. 16495913 10.1038/sj.bjc.6603023 PMC2361377

[46] Wienholds E , Plasterk RH . MicroRNA function in animal development. FEBS Lett. 2005;579(26):5911‐5922. 16111679 10.1016/j.febslet.2005.07.070

[47] Bartel DP . MicroRNAs: genomics, biogenesis, mechanism, and function. Cell. 2004;116(2):281‐297. 14744438 10.1016/s0092-8674(04)00045-5

[48] Cheng AM , Byrom MW , Shelton J , Ford LP . Antisense inhibition of human miRNAs and indications for an involvement of miRNA in cell growth and apoptosis. Nucleic Acids Res. 2005;33(4):1290‐1297. 15741182 10.1093/nar/gki200 PMC552951

[49] Ambros V . The functions of animal microRNAs. Nature. 2004;431(7006):350‐355. 15372042 10.1038/nature02871

[50] Otsuka M , Jing Q , Georgel P , et al. Hypersusceptibility to vesicular stomatitis virus infection in Dicer1‐deficient mice is due to impaired miR24 and miR93 expression. Immunity. 2007;27(1):123‐134. 17613256 10.1016/j.immuni.2007.05.014

[51] Jopling CL , Yi M , Lancaster AM , Lemon SM , Sarnow P . Modulation of hepatitis C virus RNA abundance by a liver‐specific microRNA. Science. 2005;309(5740):1577‐1581. 16141076 10.1126/science.1113329

[52] Murakami Y , Aly HH , Tajima A , et al. Regulation of the hepatitis C virus genome replication by mi R‐199a. J Hepatol. 2009;50(3):453‐460. 19144437 10.1016/j.jhep.2008.06.010

[53] Ahluwalia JK , Khan SZ , Soni K , et al. Human cellular microRNA hsa‐mi R‐29a interferes with viral nef protein expression and HIV‐1 replication. Retrovirology. 2008;5:117. 19102781 10.1186/1742-4690-5-117 PMC2635386

[54] Huang J , Wang F , Argyris E , et al. Cellular microRNAs contribute to HIV‐1 latency in resting primary CD4+ T lymphocytes. Nat Med. 2007;13(10):1241‐1247. 17906637 10.1038/nm1639

[55] Baudin F , Bach C , Cusack S , et al. Structure of influenza virus RNP. I. Influenza virsus nucleoprotein melts secondary structure in panhandle RNA and exposes the bases to the solvent. EMBO J. 1994;13(13):3158‐3165. 8039508 10.1002/j.1460-2075.1994.tb06614.x PMC395207

